# Exploring the Homeostatic and Sensory Roles of the Immune System

**DOI:** 10.3389/fimmu.2016.00125

**Published:** 2016-03-31

**Authors:** Rafael Elias Marques, Pedro Elias Marques, Rodrigo Guabiraba, Mauro Martins Teixeira

**Affiliations:** ^1^Immunopharmacology, Departamento de Bioquímica e Imunologia, Instituto de Ciências Biológicas, Universidade Federal de Minas Gerais, Belo Horizonte, Minas Gerais, Brazil; ^2^ISP, INRA, Université François Rabelais de Tours, Nouzilly, France

**Keywords:** immune system, inflammation, homeostasis, physiological role, sensing properties, information, nervous system

## Abstract

Immunology developed under the notion of the immune system exists to fight pathogens. Recently, the discovery of interactions with commensal microbiota that are essential to human health initiated a change in this old paradigm. Here, we argue that the immune system has major physiological roles extending far beyond defending the host. Immune and inflammatory responses share the core property of sensing, defining the immune system also as a sensory system. The inference with the immune system collects, interprets, and stores information, while creating an identity of self, places it in close relationship to the nervous system, which suggests that these systems may have a profound evolutionary connection.

## Bridging Inflammation, Immunology, and Physiology

Immunology comprises the study of the immune system and its functional properties, including innate and adaptive responses, immunological memory, and the relationship between the immune system and disease. The current dogma states that an immune response is induced by the recognition of molecular patterns that trigger a quick response from the innate compartment, which in turn prompts the development of adaptive immunity. This mechanism has been extensively studied in the context of infection and injury, from which current understanding of the immune system has been inferred (1). Recent discoveries demonstrating a role of the immune system in physiological contexts, including interaction with bacterial microbiota (2), in pregnancy (3), metabolism (4), organ function (5), bone homeostasis (6), exercise (7), and senescence (8), suggest that functions of the immune system extend far beyond defending the host (9). Indeed, autoimmunity (10), cancer (11), degenerative diseases (12), and psychiatric diseases (13) exemplify paradoxes and loopholes in the current understanding of the immune system, indicating its theoretical basis should be updated. There is a consensus among scientists that the ability of microorganisms or microbial products to trigger inflammation and immune responses are important for immune function. This is not being questioned here. We would like to suggest that immune functions extend far beyond interaction with pathogens.

Inflammation is a beneficial tissue response stimulated by tissue damage, which can be caused by physical, chemical, or biological stimuli (14). Inflammation is defined by the production of soluble mediators, in the alteration of vasculature, and in the recruitment of leukocytes, ultimately leading to the classical signs, such as heat, swelling, redness, pain, and loss-of-function (15, 16). Inflammation has the purpose of restoring tissue homeostasis, plays a major role in containing and resolving infection, and also occurs under sterile conditions (17). Uncontrolled inflammation can lead to further tissue damage, chronic inflammatory diseases, and autoimmunity with eventual loss of organ function (18, 19). In fact, pharmacological control of inflammation by anti-inflammatory or proresolutive compounds is effective in treating diseases, such as arthritis (20) and sepsis (21). This indicates that, during infection, targeting the host immune response instead of the causative agent itself may be an effective option in the context of disease. Thus, inflammation is prone to imbalance as many physiological machineries in the organism, and loss of balance relates to pathogenic states.

**Figure 1:**
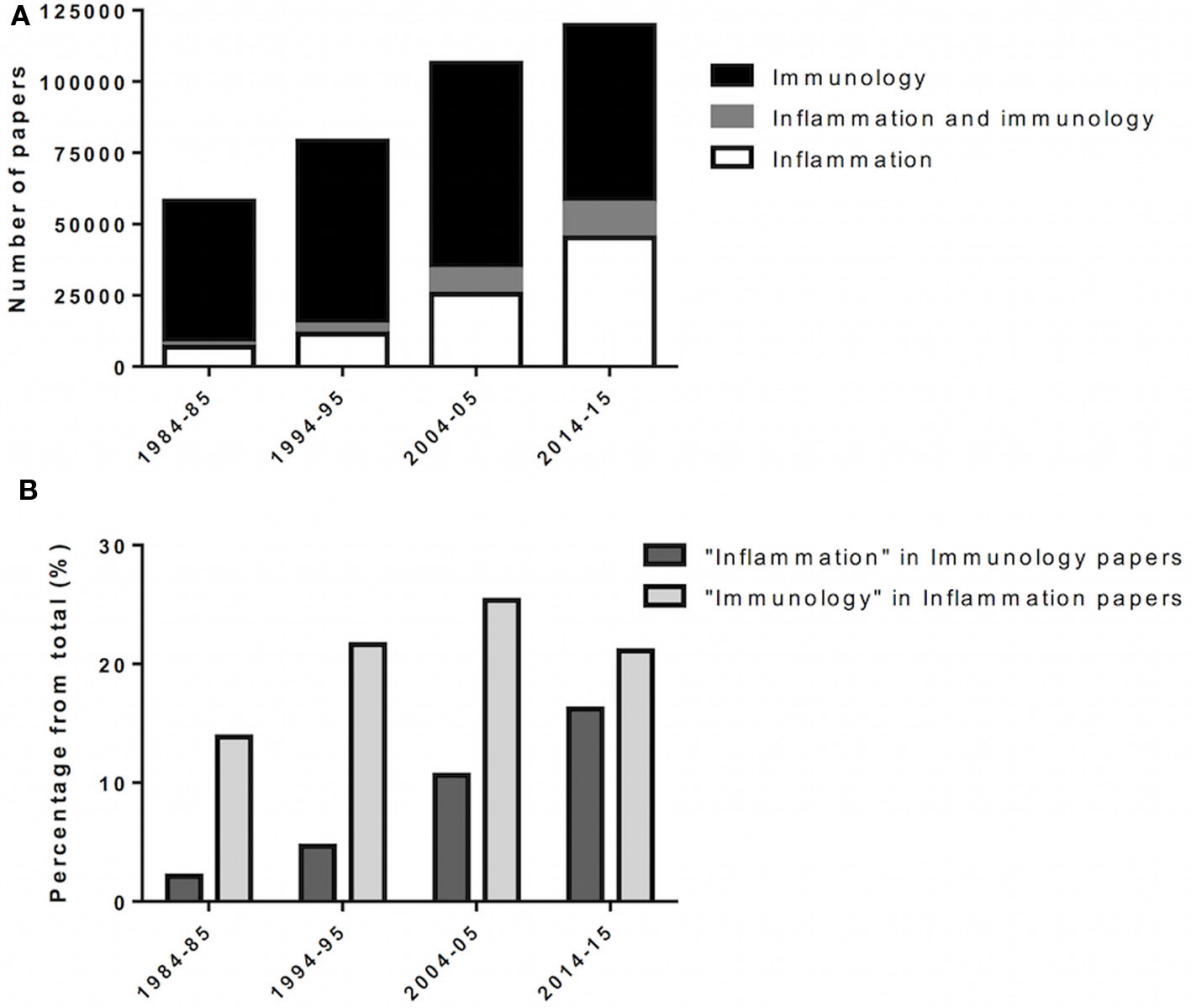
**Incorporation of inflammation into immunology**. Over the last decades, inflammation and immunology were progressively merged as biomedical research evolves. **(A)** Scientific papers, in numbers, retrieved from queries for “immunology,” “inflammation AND immunology,” and “inflammation” at PubMed (http://www.ncbi.nlm.nih.gov/pubmed). Results span 1 year (forth to fifth year) over four consecutive decades (80’ to 2010’). **(B)** Scientific papers retrieved from “immunology” for the given year/decade were queried for “inflammation,” and vice versa.

Research in biomedical sciences has shown that inflammation and immune responses coexist in the mammalian host and overlap in the context of disease (Figure 1). Although different in concept, the immune response and inflammation share biological components, e.g., leukocytes, receptors, and soluble effectors. These similarities are more pronounced when comparing inflammation to the innate immune response. In addition, it is important to consider that the immune system is in constant contact with host molecules and microorganisms, for which low-grade immune responses and inflammation may be taking place (16). In summary, we argue that inflammation and innate immunity are biological-related processes, which must operate under similar premises and toward the common goal of reaching homeostasis.

Based on the premise that inflammation share biological components with the immune response, and vice versa, the immune system must be able to respond to different stimuli. More important, this premise allows the immune system to be responsive to stimuli derived from the host cells and tissues, and not only from pathogens. This is in agreement with the “danger-sensing” paradigm and the existence of damage-associated molecular patterns (DAMPs), initially proposed by Matzinger (22). Also, studies conducted with germ-free mice have shown that these animals possess an immune system (23) and are able to inflame and to mount immune responses, though differently (24). Exposure to microbiota or to microbial products promptly restores the ability of germ-free mice to respond as conventional mice, indicating that the immune tissues and cells were present and functional in the absence of microbes. This concept of responsiveness to host leads to broader considerations, for example, that the immune system does not depend on pathogens to function or exist.

## Disease is a Rare Manifestation

**Figure 2:**
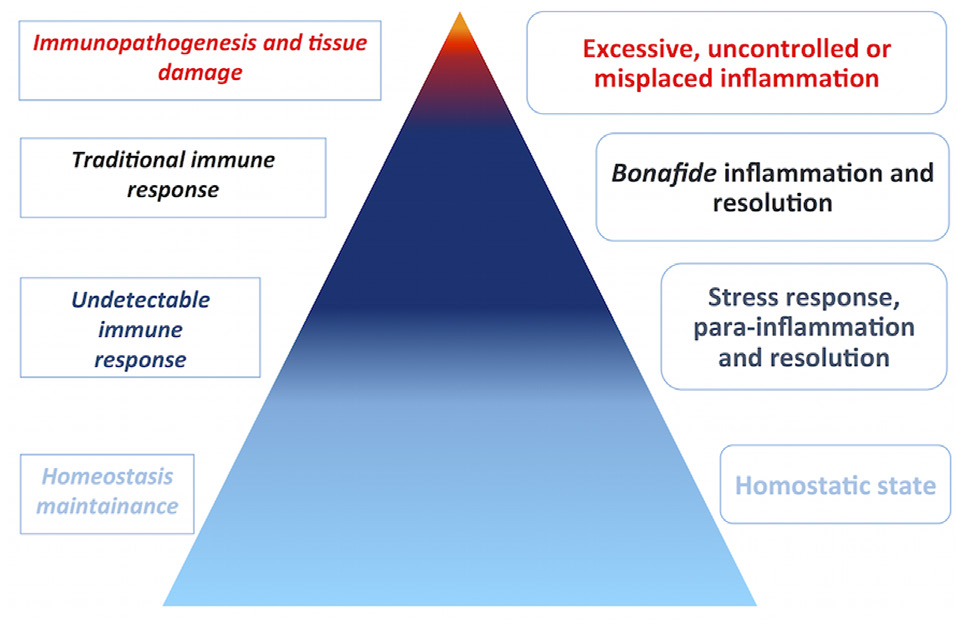
**Disease is a rare manifestation**. Biomedical research is biased toward disease, although disease does not represent homeostasis in the vertebrate host. Inflammation occurs at a graded spectrum, thus the immune system must operate in such spectrum. The pyramid estimates the proportion of immune responses that go unnoticed by current experimental techniques in cold colors, in comparison to less frequent immune responses in the context of disease, in warm colors. In the left side, immune responses are categorized by abundance and intensity, whereas, in the right side, the categories of inflammation are depicted.

According to Chovatiya and Medzhitov, inflammation is believed to occur at a graded spectrum in the vertebrate host, ranging from a homeostatic state, stress response, parainflammation, and, finally, traditional inflammation (18). The most subtle, initial inflammatory states are undetectable in light of current techniques. The immune system might therefore be considered to operate at such wide spectrum, being the immune response the extreme effort from the system to return the host to homeostasis, for example, at the onset of disease. With this in mind, one should consider that the majority of processes dealt by the immune system go unnoticed by biomedical scientists (Figure 2). The immune system has been implicated in various biological processes, indicating that it may operate together with other body systems. Recently, evidence linking the immune system to metabolism (25) and circadian cycle (26) exemplify how it is entangled in day-to-day physiological processes.

If the immune system is part of a living functioning organism, constantly reaching for homeostasis, interacting with microbiota, remodeling tissue, dealing with injury, and so forth; interacting with pathogens is a small fraction of the activities of the immune system (27). Contact with a pathogen may result in infection, and infection could lead to disease (28). Based on these possibilities and the fact that the immune system is fully active for a lifetime, disease is a rare occurrence. However, given the impact of disease in human health, it comes as no surprise that biomedical research is focused on disease, though disease does not represent or explain homeostasis between systems in a host. Although extensive knowledge on disease allows us to explore it and propose treatments, the challenge relies on studying processes that did not lead to disease, and successfully restored homeostasis.

## The Immune System as a Sensory System

The greatest challenge in reinterpreting the immune system is to define what the system is and what it does to maintain homeostasis. The basis for that may be the overlap of inflammation and immune responses. In terms of core mechanism, their common ground is sensing and recognition of molecules of variable compositions, forms, sources, and properties (14). Without sensing, the immune system is unable to respond or to sustain interaction, and similarly, inflammation is unable to start or to resolve. Interestingly, the ability of the immune system to sense things is not conflicting with sustaining interactions with both microbes and host. Therefore, we propose that the immune system is a sensory system.

**Figure 3:**
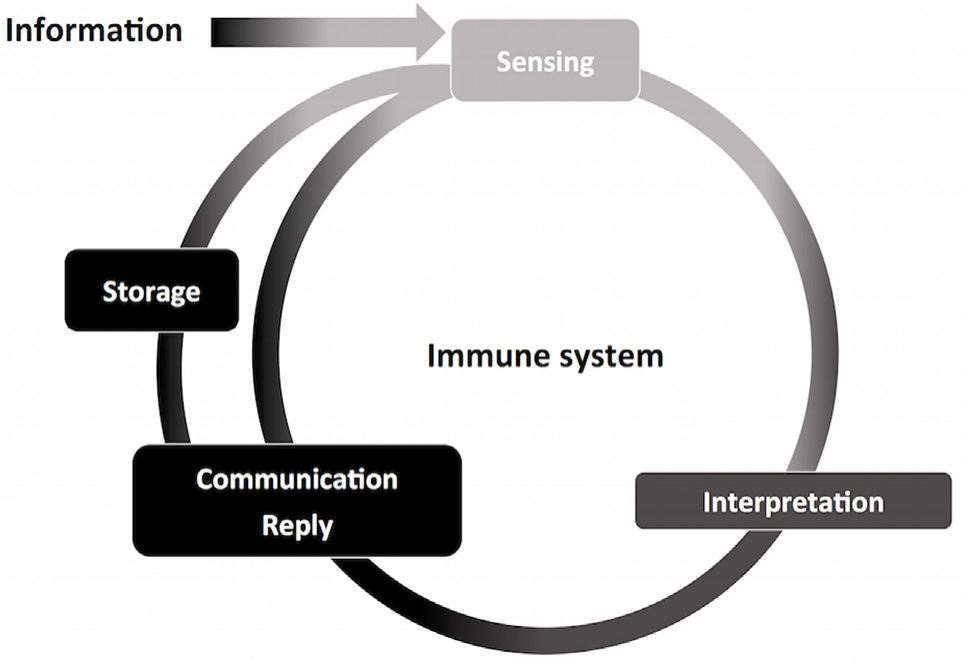
**Information management by the immune system**. The nervous and immune systems handle large volumes of information. Through information management, the immune system contributes to host homeostasis. This process start with information sensing, obtained through interaction with molecules of different origins and composition. Information is then interpreted, causing specific leukocyte activation and recruitment, and initiation of innate immunity. Finally, information is communicated and replied to other components of the immune system, such as in antigen-presenting cells and T CD4^+^ lymphocytes. The immune system is able to *store* information, for which adaptive memory is the most studied process. Stored information can be promptly accessed, exemplified by quick adaptive immune responses upon secondary challenges, adding to the ongoing flow of information through the immune system.

As any sensory system, the immune system deals with information (Figure 3). It is impossible to pinpoint a single molecular domain, macromolecule or organism that defines the sensing properties of the immune system, but interaction with all of those is translated to information. It can be detected, interpreted, stored, and replied as necessary. Also, the immune system is versatile in its interactions and powerful in reach. Microscopically, cellular compartments, organelles, vesicles, mitochondria, and the cell nucleus are crowded and constantly monitored by immune sensors (29). Macroscopically, vascular tissues are in constant scrutiny by circulating leukocytes, resident leukocytes, complement, antibodies, and the lymphatic system. These interpretations can be exemplified by the combination of signals (cytokines, metabolic intermediates, microbial products, nucleic acids, surface molecules, lipids, and antibodies) that define leukocyte recruitment, activation, and differentiation, which stands for information interpretation and reply. The hallmark of adaptive responses, immunological memory, is essentially information storage. Collectively, the bulk of information that the immune system manages is what allows the system to define and pursue homeostasis.

The ability of the immune system to interact is dependent on its ability to sense and manage information. Based on the beneficial host–microbiota interactions, McFall-Ngai proposed that adaptive immunity has evolved in part to recognize and manage complex communities of beneficial microbes (27), which confer adaptability and gain-of-function to the host, for example, in nutrient metabolism. This view is complemented by Eberl, who suggested that the immune system allows for a dynamic superorganism composed of the host and symbionts (30), and by Harvill, who added that the adaptive immunity would serve as a prospective tool for a limitless number of potential symbionts (1). In summary, they state that the immune system sustains complex relationships with beneficial bacteria that support host adaptability and homeostasis. This brings valuable perspectives on a homeostatic immune system by focusing on the host interaction with microbes. Because the immune system is entrusted with so many mechanisms of sensing (e.g., antibodies, T cell receptors, PRRs, cytokine receptors, surface molecules, and cytoplasmic molecules), there are no restraints to what can be sensed and from which source. Thus, it is likely that the immune system mediates both intra- and inter-organisms interactions. The immune system would provide flexibility to the host when dealing with the environment and with itself, consequently adding flexibility to the management of homeostasis. For example, the immune system participates in glucose metabolism, even though glucose metabolism is ancient and evolutionary conserved. Leukocytes express isoforms of GLUT and are sensitive to variations in glucose levels (31–33). Leukocytes are activated by adipokines (e.g., leptin) (34) and have been implicated in insulin resistance (35–37). Also, inflammasome activation was reported alongside insulin secretion in pancreatic beta cells (33). Altogether, those examples corroborate our hypothesis by showing how specialized the immune system is to sense and manage alterations in the host homeostasis (e.g., metabolism), over other body systems not directly involved in sensing.

On the other hand, in case of intrinsic failure of the immune system, a simple stimulus could lead to disastrous consequences, such as widespread infection, shock, or cancer. Inferring that the immune system plays an important role in sustaining intrahost interactions would also explain why immune responses and inflammation have great influence over other host systems. Therefore, immunity can be described as a host system of information management, which allows all possible interactions the host may have to sustain, provides host cohesion, adaptability, thus increasing host fitness.

## Information Management in the Immune and Nervous Systems

The nervous system is traditionally associated with sensing and management of information in the vertebrate host. The suggestion that the primary function of the immune system is also sensing and management of information may be conflicting, since these are already entrusted to another evolutionary-conserved body system. Moreover, sensing is a general property of multicellular organisms and essential for host fitness. Although it may appear controversial to have two complex systems in charge of the same function, which already exists in the host, the nervous and immune systems are not redundant. These systems mediate interactions between host and environment that exceed interactions made by any other body systems in number and complexity. The nervous and immune systems share remarkable similarities, such as full coverage of the host and interactivity, but are diversified in niche. The nervous system is responsible for sensing physical stimuli, which we understand as sight, hearing, taste, touch, and handling of autonomic functions (e.g., breathing, blood pressure, and digestion). This panel is complemented by the immune system’s sensing of chemical stimuli, such as biological macromolecules, microbial components, metabolites, and soluble mediators, such as cytokines and antibodies. Finally, both systems confer a notion of “self” to the host, by defining what the host is from different perspectives. The notion of self is a key aspect for interaction between the host and other organisms.

The immune and nervous systems deal with information in a similar way, by sustaining specific connections between living cells in a given time and space. This concept is easily exemplified by the neuronal synapse, which has been imaged at great resolution recently (38). The immune system has its own counterpart, the immunological synapse, mostly exemplified by the interaction between lymphocytes and antigen-presenting cells. Interestingly, the immune synapse also involves the analogous release of microvesicles into the synaptic cleft, but in this case loaded with T-cell receptors (39). Also, as the nervous system has major areas for information processing and transmission [ganglia and central nervous system (CNS)], the immune system has lymph nodes and lymphoid organs (thymus and spleen). In this line, memory lymphocytes acquire a long-lived phenotype in order to store information, which is a main property of neurons. Finally, complexity may be considered a common feature between both systems. They probably contain the larger number of unique cells, i.e., every single neuron or lymphocyte is different from all the others in the body. Immune and nervous systems are considered to contain around 10^11^ cells, which might interact with several other cells simultaneously (38). Consequently, these systems could manage immeasurable amounts of information and interaction in vertebrate organisms regularly.

## Interaction and Evolution of Immune and Nervous Systems

Recent studies indicate that the nervous and immune systems interact extensively (40). Louveau and colleagues described a lymphatic system within the CNS that allows for lymphatic drainage and leukocyte trafficking (41), contradicting the assumption that the CNS is an immune-privileged site. Moreover, the CNS is populated by its own resident macrophage-like cells, the microglia, which participates in inflammation and immune responses (42). Likewise, immune tissues are innervated and subject to neurotransmitter stimuli, modulating leukocyte activity (43). Expression of neurotransmitters and neurotransmitter receptors was demonstrated in macrophages and lymphocytes (44), which allows for direct communication between leukocytes and neurons. In addition, Kim and colleagues recently found that tumor necrosis factor (TNF) acts in the brain during bacterial infection to increase adaptive immune responses. This increase was mediated by hypothalamic induction of lipolysis, exemplifying how the nervous system, immune system, and metabolism are intertwined (45).

Interactions between the nervous and immune systems are bidirectional, allowing immune responses to influence vertebrate behavior and cognitive functions, and vice versa (46). Stress is known to cause immune suppressive effects, which in turn affects human behavior by insufficient glucocorticoid signaling leading to stress-related neuropsychiatric disorders (47). Stress-induced immune dysregulation has a huge impact on the host, as it reduces the effectiveness of vaccines, slows wound healing, reactivates latent viral infections, and increases susceptibility to severe diseases (46). Immune dysfunction is frequently observed in patients suffering from psychiatric diseases and is characterized by low-grade systemic inflammation. This phenomenon consists in an increase in circulating levels of cytokines and in changes in the proportions of circulating leukocytes and their degree of activation (48, 49). Low-grade systemic inflammation is associated with aging, and it has been described in patients with obsessive-compulsive disorder, major depression, bipolar disorder, Parkinson disease, and Alzheimer disease (50–53). Interleukin-6 (IL-6) was found to be increased systemically in patients suffering from these psychological diseases and correlates with the finding that old individuals that express more IL-6 develop cognitive decline more frequently (54). These findings indicate that the immune system detects homeostatic imbalances in the nervous system and is influenced by these imbalances, suggesting that homeostasis and function of immune and nervous systems are largely dependent on each other. The idea that behavior and cognition have an impact in immunity is not widespread among immunologists, but nonetheless it should be taken into consideration. Pain is mediated by the joint action of nervous and immune systems and exemplifies how immune responses directly affect human behavior, notably mood (55, 56).

The abundance of data supporting the high level of interaction between nervous and immune system is also suggestive of a close evolutionary connection. Niche diversification suggests that the vertebrate nervous and immune systems may have shared a common neuroimmune ancestor or that the immune system derived from the nervous system. A defining feature of vertebrates is the emergence of new embryonic cell types: (I) neural crest, which originates from within the developing CNS and is able to migrate and differentiate into many tissues, such as bone, cartilage, neurons, and glia (57), and (II) neurogenic placodes, which give rise to sensory neurons that ultimately compose vertebrates’ sensory systems (58). Neural crest and neurogenic placodal cells are responsible for the emergence of the vertebrate head, which accommodates the CNS, and later the jaw. As pointed by Diogo and colleagues, the new head required muscles and a new heart, for which head muscles and a powerful chambered heart evolved (58). Together, these changes are synchronized to the appearance of the vertebrate immune system, in a “boom” of diversification and gain-of-function, exemplified by novel molecules, cell types, and adaptive immunity (59). In a remarkable coincidence, the existence of bone and bone marrow would support leukocyte replication and maturation, whereas a novel circulatory system would distribute these leukocytes to the entire host, in the context of increased complexity of interaction between host systems and between host and microbiota, as ingestion habits changed in jawed vertebrates. Such conditions not only nurture the evolution of a sensing/interacting system but also require it for homeostasis of the host. Therefore, it is likely that the neural crest resulted in development of the immune system in vertebrates and brought the nervous and immune systems together, from an evolutionary perspective. Of note, jawed vertebrates comprise 99% of living vertebrate species, including humans, demonstrating the evolutionary importance of these events (60). This evolutionary connection is supported by Hepburn and colleagues, who identified nerve growth factor β as an equivalent to *Drosophila* toll ligand Spätzle in chordates, which is associated with immune responses to bacteria (61).

## Final Remarks

The understanding of the immune system has changed drastically in the last decade (62). A homeostatic paradigm of immunity is already accepted by a significant part of the scientific community, and new ideas, such as disease tolerance (9), add interesting perspectives to the field. Accordingly, recent findings suggest the immune system also maintain virus–host interactions (63). Although great advances have been made in interpreting the roles of immunity from a homeostatic perspective unbiased by pathogens, thorough research and thinking are still needed. The complexity of the immune system is only matched by its ability to sustain greater complexity. As the immune system role in supporting host–microbiota interactions was consolidated, scientists now turn to the role of the immune system in other biological processes or systems, such as metabolism and the nervous system. The field of neuroimmunology shows promise, as the immune and nervous systems seem intimately related in function, interaction, and evolution. Pain is a connecting point between neurology and immunology where sensing and interactive properties of nervous and immune systems converge. Thus, pain translates into a great opportunity for research.

## Author Contributions

RM conceived and wrote the manuscript. PM, RG, and MT contributed with both intellectual and written information. RM, PM, and RG prepared figures. All authors agreed on the final version of the manuscript and are accountable for its contents.

## Conflict of Interest Statement

The authors declare that the research was conducted in the absence of any commercial or financial relationships that could be construed as a potential conflict of interest.
